# First wave of COVID-19 hospital admissions in Denmark: a Nationwide population-based cohort study

**DOI:** 10.1186/s12879-020-05717-w

**Published:** 2021-01-09

**Authors:** Jon Gitz Holler, Robert Eriksson, Tomas Østergaard Jensen, Maarten van Wijhe, Thea Kølsen Fischer, Ole Schmeltz Søgaard, Simone Bastrup Israelsen, Rajesh Mohey, Thilde Fabricius, Frederik Jøhnk, Lothar Wiese, Stine Johnsen, Christian Søborg, Henrik Nielsen, Ole Kirk, Birgitte Lindegaard Madsen, Zitta Barrella Harboe

**Affiliations:** 1 Department of Pulmonary and Infectious Diseases, University Hospital of Copenhagen, North Zealand Hospital, Hillerød, Denmark; 2 Department of Disease Systems Biology, Novo Nordisk Foundation Center for Protein Research, University of Copenhagen, Copenhagen, Denmark; 3 Department of Science and Environment, Roskilde University, Roskilde, Denmark; 4 University of Southern Denmark, Odense, Denmark; 5 Department of Infectious Diseases, Aarhus University Hospital, Aarhus, Denmark; 6 Department of Infectious Diseases, Copenhagen University Hospital, Hvidovre, Denmark; 7 Department of Internal Medicine, Aarhus University Hospital, Herning, Denmark; 8 Department of Infectious Diseases, University Hospital of Southern Denmark, Odense, Denmark; 9 Department of Infectious Diseases, Lillebaelt Hospital, Vejle, Denmark; 10 Department of Infectious Diseases, Zealand University Hospital, Roskilde, Denmark; 11 Department of Pulmonary and Infectious Diseases, Copenhagen University Hospital, Bispebjerg, Denmark; 12 Department of Infectious Diseases, Copenhagen University Hospital, and Gentofte, Herlev, Denmark; 13 Department of Infectious Diseases, Aalborg University Hospital, Aalborg, Denmark; 14 Department of Infectious Diseases, Rigshospitalet, Copenhagen, Denmark; 15 Centre for Physical Activity, Rigshospitalet, University of Copenhagen, Copenhagen, Denmark

**Keywords:** SARS-CoV-2, COVID-19, Prognostic factors, Mortality, Nationwide, Intensive care unit, Epidemiology

## Abstract

**Background:**

Severe acute respiratory syndrome coronavirus 2 (SARS-CoV-2) and its associated disease coronavirus disease 2019 (COVID-19), is a worldwide emergency. Demographic, comorbidity and laboratory determinants of death and of ICU admission were explored in all Danish hospitalised patients.

**Methods:**

National health registries were used to identify all hospitalized patients with a COVID-19 diagnosis. We obtained demographics, Charlson Comorbidity Index (CCI), and laboratory results on admission and explored prognostic factors for death using multivariate Cox proportional hazard regression and competing risk survival analysis.

**Results:**

Among 2431 hospitalised patients with COVID-19 between February 27 and July 8 (median age 69 years [IQR 53–80], 54.1% males), 359 (14.8%) needed admission to an intensive care unit (ICU) and 455 (18.7%) died within 30 days of follow-up. The seven-day cumulative incidence of ICU admission was lower for females (7.9%) than for males (16.7%), (*p* < 0.001). Age, high CCI, elevated C-reactive protein (CRP), ferritin, D-dimer, lactate dehydrogenase (LDH), urea, creatinine, lymphopenia, neutrophilia and thrombocytopenia within ±24-h of admission were independently associated with death within the first week in the multivariate analysis. Conditional upon surviving the first week, male sex, age, high CCI, elevated CRP, LDH, creatinine, urea and neutrophil count were independently associated with death within 30 days. Males presented with more pronounced laboratory abnormalities on admission.

**Conclusions:**

Advanced age, male sex, comorbidity, higher levels of systemic inflammation and cell-turnover were independent factors for mortality. Age was the strongest predictor for death, moderate to high level of comorbidity were associated with a nearly two-fold increase in mortality. Mortality was significantly higher in males after surviving the first week.

**Supplementary Information:**

The online version contains supplementary material available at 10.1186/s12879-020-05717-w.

## Background

Coronavirus disease 2019 (COVID-19), caused by Severe acute respiratory syndrome coronavirus 2 (SARS-CoV-2), is a global emergency. The first case in Denmark was diagnosed on February 27th, 2020, and during the first month, the number of laboratory-confirmed cases rose to more than 3000; close to 500 patients were hospitalised, of which 30% required admission to an intensive care unit (ICU) and 5% died (www.sst.dk/corona). Starting on March 13th, Denmark introduced comprehensive lockdown measures, as one of the first European countries, and four weeks later the country gradually reopened. By early July, the daily incidence of new COVID-19 cases was below 0.35 per 100.000 marking the end of the first wave of the epidemic in Denmark (www.sst.dk/corona).

Despite variations reported across countries, emerging evidence consistently indicates that older men with multiple comorbidities have poorer COVID-19-related outcomes [1–3]. To date, studies from Denmark focusing on the overall epidemiological characteristics during the early phase of the epidemic [4, 5] and the association with several cardiovascular outcomes have been made publicly available [6]. From these early reports, COVID-19 related mortality in Denmark have been described to vary between 9.3 and 16.6% and rates of ICU admission between 12.9 and 17.8% depending on the specific groups of patients studied [5, 6].

Differences in laboratory markers of newly admitted SARS-CoV-2 positive cases and their association to severe outcomes have been reported from previous studies [3, 7–17], but remain unexplored in Denmark and other Scandinavian populations. We present a complete nationwide observational study with comprehensive clinical and laboratory parameters during the first five months of the COVID-19 epidemic in Denmark. The aim was to study demographic, comorbidity and laboratory determinants of death and ICU admission in Denmark.

## Methods

### Study design, setting and participants

All residents of Denmark have free access to health care. Using the Danish National Patient Register (DNPR), which contains detailed information on all hospital contacts nationwide, we identified all individuals in the entire population of 5.8 million [18] who had received a COVID-19 diagnosis (https://www.who.int/classifications/icd/covid19/en). Diagnoses in DNPR are coded according to the International Classification of Diseases, 10th revision (ICD-10). All hospital contacts between January 1 and July 8 with an ICD-10 diagnosis code of *COVID-19 severe acute respiratory syndrome* (B972A) and/or *COVID-19 infection unspecified sites* (B342A) regardless of duration were considered eligible for inclusion.

The codes ‘U07.1 COVID-19, virus identified’ and ‘U07.2 COVID-19, virus not identified’ as suggested by The World health Organization were implemented by The Danish Health Data Authority on 1 July 2020 in order to align with international coding standards and were therefore not available during the study period. Patients without a unique personal registration number (PRN) in Denmark were not present.

We defined a hospital admission as any hospital contact that lasted more than 12 h or a shorter contact which resulted in transfer to an ICU and/or death. Several hospital contacts within 24 h were considered as one event. When admissions were less than one hour apart, the patient was considered to be hospitalised the period in between as well, thus counting towards the 12-h hospital stay. The start of the first hospital admission to which the first COVID-19 diagnosis could be linked and which was not more than 14 days before or 30 days after the date of first COVID-19 diagnosis, was defined as the index date and was used to determine baseline characteristics as well as the start of follow-up (77.2% were admitted on the day of diagnosis).

All COVID-19 diagnoses were based on a positive polymerase chain reaction (PCR) test for SARS-CoV-2 on respiratory samples collected by naso- or oropharyngeal swab, sputum expectoration, or tracheal suction.

### Data sources and collection

The Danish Civil Registration System (CRS) assigns all residents a unique PRN which enables accurate linkage between the Danish national registries [19, 20]. We extracted information from the following specific registries: 1) The CRS [20], which comprise date of birth, sex, vital status, date of death, emigration, area of residence; 2) The DNPR [21], which includes dates of admission and discharge, admitting departments, and all primary and secondary discharge diagnoses and procedure codes from hospital contacts; 3) Register of Laboratory Results for Research (RLRR) [19], which contains nationwide laboratory information (except Midtjylland Region, population 1.3 million) using Nomenclature for Property and Unit (NPU) codes (13). Subsequent analyses were performed on pseudo anonymized data.

### Variables and outcomes

The primary outcomes were death within 7 and 30 days from the index date. Need for ICU admission (including admission to an ICU ward or the codes for use of invasive mechanical ventilation (IMV), dialysis, inotropic/vasopressor therapy and extracorporeal membrane oxygenation (ECMO) (http://medinfo.dk/sks/brows.php), were used as secondary outcomes (Supplementary Table S1). Age, sex, underlying medical conditions, and laboratory parameters were included in the analysis. We calculated the Charlson comorbidity index modified by Quan (CCI) at time of admission, by retrieving ICD-10 coded hospital discharge diagnoses from the previous 10 years, and categorized patients with low (0), moderate (1–2) or high (> 2) levels of comorbidity [22].

Laboratory parameters were collected from admitted patients within ±24 h of the index date. If a measurement was repeated within this period, we used an average. Patients were followed from the index date until the date of death, completion of 30 days of follow-up, or July 82,020, whichever first.

### Statistical analysis

Baseline characteristics are presented as medians with interquartile range (IQR) for continuous variables, and numbers and percentages for categorical variables. All-cause 30-day mortality stratified by sex, age intervals, and CCI were investigated with Kaplan-Meier (KM) analyses. Prognostic factors for the primary outcomes were evaluated in a univariate and multivariate Cox proportional hazard regression and presented as unadjusted and adjusted hazard ratios (HRs) with 95% confidence intervals (CI). Covariates included age, sex, CCI and laboratory parameters (when available). Interaction between covariates where examined. Cumulative incidence rates of ICU admission were analysed with competing risk survival analysis (Aalen Johansen estimator with death as a competing event) using Grays test for statistical significance. Laboratory parameters underwent additional univariate analysis for differences between subgroups, using t-test for statistical significance. The analyses were stratified by sex, and primary and secondary outcomes.

Statistical significance level was set at 0.05. All tests of significance were two-sided. Statistical analyses were performed using Stata (Stata Corporation LP, Texas, USA) or R (R Foundation for Statistical Computing, Vienna, Austria). The study was conducted according to the STROBE statement [23].

## Results

### Patients

We identified 4981 hospital contacts with a COVID-19 diagnosis, of these 2465 were hospitalised, and 2431 met our inclusion criteria. The median age of hospitalised patients was 69 years (IQR 53–80), the median length-of-stay was 5.5 days (IQR 2.2–11.1) and 54.1% were male. On admission, 60.7% had a registered comorbidity within the past 10 years. The most common comorbidities were hypertension (*n* = 412, 16.9%), immunosuppression and cancer (*n* = 459, 18.9%), and diabetes (*n* = 315, 13.0%). In total, 1440 (59.2%) of the hospitalised patients had a CCI of zero (Table 1).

**Table 1:** Baseline characteristics of admitted COVID-19 diagnosed patients

	Admitted	Admitted to ICU	30-day follow-up	
**Variable**	**Total**		**Survivors**	**Non-survivors**
**N (%)**	2431	359 (14.8%)	1976 (81.3%)	455 (18.7%)
**Age in years, Median (IQR, 25–75%)**	69 (53–80)	69 (59–75)	64 (49–77)	81 (74–86.5)
**Sex (%)**				
Female	1116 (45.9%)	106 (29.5%)	(47.5%)	178 (39.1%)
Male	1315 (54.1%)	253 (70.5%)	1038 (52.5%)	277 (60.9%)
**Age in age groups, year (%)**				
0–64	1039 (42.7%)	135 (37.6%)	1016 (51.4%)	23 (5.1%)
65–84	1072 (44.1%)	212 (59.1%)	790 (40.0%)	282 (62.0%)
85+	320 (13.2%)	12 (3.34%)	170 (8.6%)	150 (33.0%)
**Admissions and duration**				
Median duration of hospitalizations > 12 h in days (IQR)	5.47 (2.2–11.11)	19.38 (10.42–29.05)	5.19 (1.6–10.55)	7.02 (3.35–11.48)
Patients needing				
ICU admission	359 (14.8%)	359 (100%)	252 (12.8%)	107 (23.5%)
Mechanical respirator use	240 (9.9%)	240 (66.9%)	167 (8.5%)	73 (16.0%)
ECMO	18 (0.7%)	18 (5%)	13 (0.7%)	5 (1.1%)
Dialysis	78 (3.2%)	78 (21.7%)	48 (2.4%)	30 (6.6%)
Inotropic/Vasopressor use	210 (8.6%)	210 (58.5%)	143 (7.2%)	67 (14.7%)
Comorbidities***				
**Any comorbidities**	1475 (60.7%)	225 (62.7%)	1086 (55.0%)	389 (85.5%)
**Cardiovascular disease**				
Myocardial infarct	122 (5%)	22 (6.1%)	83 (4.2%)	39 (8.6%)
Hypertension	412 (16.9%)	55 (15.3%)	294 (14.9%)	118 (25.9%)
Congestive heart failure	175 (7.2%)	19 (5.3%)	114 (5.8%)	61 (13.4%)
Peripheral vascular disease	144 (5.9%)	30 (8.4%)	93 (4.7%)	51 (11.2%)
Cerebrovascular diseases	259 (10.7%)	28 (7.8%)	176 (8.9%)	83 (18.2%)
**Chronic respiratory disease**	298 (12.3%)	45 (12.5%)	219 (11.1%)	79 (17.4%)
Asthma	122 (5.0%)	20 (5.6%)	108 (5.5%)	14 (3.1%)
COPD	216 (8.9%)	36 (10.0%)	143 (7.2%)	73 (16%)
**Immunosuppression**	459 (18.9%)	84 (23.4%)	327 (16.5%)	132 (29%)
Of which cancer	324 (13.3%)	64 (17.8%)	234 (11.8%)	90 (19.8%)
**Kidney disease**	130 (5.3%)	22 (6.1%)	79 (4.0%)	51 (11.2%)
**Rheumatologic disease/Connective tissue disease**	104 (4.3%)	17 (4.7%)	78 (3.9%)	26 (5.7%)
**Liver disease**	51 (2.1%)	10 (2.8%)	43 (2.2%)	8 (1.8%)
**Metabolic disease**				
Diabetes	315 (13.0%)	55 (15.3%)	225 (11.4%)	90 (19.8%)
Obesity	135 (5.6%)	23 (6.4%)	112 (5.7%)	23 (5.1%)
**Neurological disease**	271 (11.1%)	38 (10.6%)	206 (10.4%)	65 (14.3%)
**Comorbidity level (CCI)**				
0 (reference)	1440 (59.2%)	201 (56.0%)	1281 (64.8%)	159 (34.9%)
1 to 2	703 (28.9%)	118 (32.9%)	512 (25.9%)	191 (42.0%)
> 2	287 (11.8%)	40 (11.1%)	182 (9.2%)	105 (23.1%)

Groups with less than five patients are presented as < 5

* Values expressed as total number (fraction) and medians [25 percentile-75 percentile] as appropriate. Chi-squared test for categorical variables and Kruskal-Wallis test for continuous variables

**Readmission is defined as a hospital contact at least 24 h after a previous hospital contact and a duration of 12 h or more

*** Any hospital contacts in the past 10 years

### Laboratory parameters

Laboratory parameters within 24 h of admission were available from 1999 patients (82%). Stratified univariate analyses of laboratory parameters at the time of hospitalisation revealed significant sex differences in levels of C-reactive protein (CRP), ferritin, lymphocyte and platelet counts, lactate dehydrogenase (LDH), creatinine, urea, and blood glucose. Males presented with more pronounced laboratory abnormalities on admission, compared to females, but differences were less pronounced in the subgroups of patients who later required ICU admission or those who died (Table 2). Patients who needed ICU admission or died, had more marked derangement of all the above test results (Table 3).

**Table 2:** Laboratory results from all COVID-19 hospitalised patients at admission by registered sex, stratified on all admitted patients, patients admitted to an ICU, and non-survivors. Only the first contact contributed. Multiple laboratory systems are used in the Danish healthcare system to analyse biochemical samples. These systems may be used with model adapted reference ranges. In this table the reference ranges are medians of all the reporting laboratories and as a result the reference ranges are only intended as interpretation assistance. Groups with less than five patients are presented as < 5. *P*-values< 0.05 are bold

Laboratory parameters (unit)	Median reference interval	Baseline biochemistry for all admitted patients					Baseline biochemistry for patients later admitted to an ICU					Baseline biochemistry for non-survivors in the follow-up period				
		Male ***n*** = 1315		Female ***n*** = 1116			Male ***n*** = 212		Female ***n*** = 84			Male ***n*** = 244		Female ***n*** = 161		
		n (%)	median (ICR)	n (%)	median (ICR)	p-value	n (%)	median (ICR)	n (%)	median (ICR)	p-value	n (%)	median (ICR)	n (%)	median (ICR)	p-value
**Acute phase reactants**																
C-reactive protein (mg/L)	< 7	960 (73)	73.25 (35.50–130.00)	813 (73)	50.00 (21.10–99.50)	**< 0.001**	200 (79)	120.67 (64.13–190.00)	71 (67)	78.67 (39.00–127.08)	**0.0025**	235 (85)	94.00 (49.75–154.00)	157 (88)	74.50 (40.00–130.00)	0.13
Ferritin (μg/L)	15–245	320 (24)	730.50 (332.21–1426.50)	269 (24)	284.67 (137.50–577.00)	**< 0.001**	66 (26)	1160.00 (622.38–1990.00)	20 (19)	646.00 (372.63–1137.25)	**0.011**	70 (25)	710.00 (314.50–1474.50)	44 (25)	329.00 (161.13–709.25)	**0.015**
Procalcitonin (μg/L)	< 0.30	200 (15)	0.20 (0.09–0.60)	141 (13)	0.08 (0.05–0.22)	0.16	62 (25)	0.46 (0.20–1.35)	17 (16)	0.23 (0.14–0.62)	0.18	40 (14)	0.58 (0.17–1.41)	19 (11)	0.16 (0.10–0.35)	**0.010**
Erythrocyte sedimentation rate (mm)	2.0–17.5	< 5		6 (1)	38.50 (16.25–55.50)		< 5		< 5			< 5		< 5		
Haptoglobin (g/L)	0.35–2.05	9 (1)	2.83 (1.69–3.50)	13 (1)	1.84 (1.59–4.02)	0.97	< 5		< 5			< 5		< 5		
**Haematology**																
Haemoglobin (mmol/L)	6.60–9.65	955 (73)	8.32 (7.45–9.05)	815 (73)	7.83 (7.10–8.40)	**< 0.001**	202 (80)	8.30 (7.65–9.06)	73 (69)	7.74 (6.88–8.40)	**< 0.001**	230 (83)	7.90 (6.70–8.73)	154 (87)	7.69 (6.58–8.26)	0.16
White blood cell count (× 10^9^/L)	4.45–13.30	959 (73)	6.65 (5.05–9.06)	810 (73)	6.14 (4.60–8.60)	0.28	201 (79)	7.30 (5.30–10.20)	71 (67)	6.30 (4.63–8.43)	0.12	233 (84)	7.05 (5.35–9.90)	157 (88)	7.75 (5.30–10.70)	0.58
Lymphocyte count (× 10^9^/L)	1.20–4.00	870 (66)	0.92 (0.70–1.30)	753 (67)	1.05 (0.75–1.41)	**0.029**	179 (71)	0.83 (0.61–1.10)	65 (61)	0.84 (0.60–1.13)	0.89	209 (75)	0.80 (0.60–1.13)	139 (78)	0.90 (0.63–1.30)	0.67
Neutrophil count (× 10^9^/L)	1.60–7.10	701 (53)	4.90 (3.50–7.25)	579 (52)	4.50 (2.95–6.55)	0.13	139 (55)	5.96 (4.20–8.53)	51 (48)	5.30 (3.57–7.10)	0.084	165 (60)	5.45 (3.75–8.17)	112 (63)	6.60 (3.98–8.63)	0.40
Monocyte count (× 10^9^/L)	0.20–1.20	870 (66)	0.50 (0.30–0.70)	753 (67)	0.50 (0.30–0.70)	0.59	179 (71)	0.40 (0.25–0.56)	65 (61)	0.40 (0.23–0.60)	0.72	209 (75)	0.45 (0.30–0.70)	139 (78)	0.50 (0.31–0.80)	0.11
Basophil count (× 10^9^/L)	0.00–0.20	870 (66)	0.00 (0.00–0.05)	753 (67)	0.00 (0.00–0.05)	0.96	179 (71)	0.00 (0.00–0.05)	65 (61)	0.00 (0.00–0.05)	0.46	209 (75)	0.00 (0.00–0.03)	139 (78)	0.00 (0.00–0.03)	0.71
Platelet count (× 10^9^/L)	145–555	932 (71)	198.00 (153.00–250.13)	802 (72)	217.50 (171.00–290.75)	**< 0.001**	199 (79)	200.00 (152.25–263.33)	73 (69)	197.50 (150.50–270.50)	0.47	225 (81)	189.00 (140.00–242.00)	154 (87)	217.75 (159.13–299.75)	**0.0029**
**Organ-specific markers**																
Alanine transaminase (U/L)	8–45	889 (68)	32.00 (22.00–52.00)	754 (68)	24.50 (17.50–39.00)	**0.044**	188 (74)	37.50 (26.00–66.25)	67 (63)	26.00 (20.00–39.58)	0.59	214 (77)	29.50 (19.25–47.63)	139 (78)	22.00 (16.00–33.00)	0.92
Aspartate transaminase (U/L)	15–40	39 (3)	46.00 (30.00–69.25)	31 (3)	32.00 (24.00–49.50)	0.94	18 (7)	61.75 (43.00–74.50)	< 5			6 (2)	50.50 (27.50–82.50)	< 5		
Bilirubin (μmol/L)	3.40–21.50	891 (68)	9.25 (7.00–13.00)	759 (68)	7.00 (5.00–10.00)	**< 0.001**	188 (74)	9.90 (7.00–13.50)	67 (63)	7.60 (6.40–11.00)	0.58	218 (79)	9.55 (6.57–13.00)	140 (79)	7.83 (5.00–10.13)	0.99
Amylase (U/L)	25–112.5	447 (34)	60.00 (42.75–82.00)	382 (34)	56.00 (41.00–78.00)	0.10	96 (38)	54.75 (41.00–79.13)	36 (34)	55.00 (36.13–96.88)	0.39	120 (43)	66.33 (43.00–93.00)	69 (39)	61.00 (40.00–88.00)	0.39
Alkaline phosphatase (U/L)	50–284	838 (64)	68.58 (55.00–94.00)	718 (64)	76.00 (59.00–97.00)	0.54	171 (68)	67.00 (52.00–96.00)	57 (54)	66.00 (52.00–110.00)	0.84	208 (75)	74.00 (57.00–115.50)	134 (75)	88.50 (67.00–111.50)	0.29
Lactate dehydrogenase (U/L)	138–271	817 (62)	288.50 (217.00–382.00)	689 (62)	247.00 (198.50–330.00)	**< 0.001**	166 (66)	400.00 (306.75–548.42)	62 (58)	336.67 (231.38–528.13)	0.16	198 (71)	302.50 (231.63–436.25)	123 (69)	270.00 (224.50–380.00)	**0.010**
Troponin I (ng/L)	< 45	86 (7)	19.25 (11.03–39.00)	78 (7)	14.68 (7.10–44.08)	0.31	24 (9)	31.00 (13.68–60.38)	12 (11)	25.40 (13.69–68.75)	0.97	12 (4)	31.60 (11.95–58.25)	15 (8)	50.00 (34.00–135.50)	0.14
Creatine kinase [muscle brain] (μg/L)	0.00–5.50	61 (5)	1.10 (1.00–2.80)	48 (4)	1.00 (1.00–2.00)	0.94	13 (5)	2.30 (1.30–3.00)	6 (6)	1.00 (1.00–1.23)	0.23	11 (4)	3.00 (1.55–7.85)	11 (6)	2.00 (1.00–4.30)	0.68
Creatine kinase (U/L)	40–260	151 (11)	150.00 (84.75–463.00)	93 (8)	80.00 (48.00–160.00)	**< 0.001**	31 (12)	257.00 (133.00–854.00)	5 (5)	126.00 (38.00–144.50)	**0.039**	32 (12)	220.00 (99.63–744.50)	17 (10)	84.00 (32.50–127.00)	**0.0044**
Thyroid-stimulating hormone (10^−3^ IU/L)	0.55–4.65	190 (14)	1.10 (0.70–1.74)	174 (16)	1.30 (0.71–2.12)	0.80	33 (13)	1.03 (0.43–1.53)	17 (16)	0.93 (0.66–1.66)	0.56	42 (15)	1.05 (0.69–1.92)	31 (17)	1.50 (0.80–1.90)	0.43
Creatinine (μmol/L)	36.50–62.00	783 (60)	92.50 (77.00–120.00)	706 (63)	68.88 (57.00–88.00)	**< 0.001**	158 (62)	99.75 (82.38–125.00)	62 (58)	82.50 (65.63–113.25)	0.28	204 (74)	112.00 (89.25–157.50)	144 (81)	80.75 (65.50–124.08)	**< 0.001**
Glomerular filtration rate (estimated) (mL/min)	> 59.50	956 (73)	72.00 (51.00–88.00)	809 (72)	77.00 (55.00–90.00)	**0.0094**	201 (79)	67.50 (52.50–85.50)	72 (68)	64.25 (51.25–82.75)	0.34	235 (85)	52.50 (35.17–71.25)	157 (88)	59.00 (36.00–76.00)	0.32
Urea (mmol/L)	2.60–7.50	844 (64)	6.93 (5.00–10.26)	715 (64)	5.70 (3.80–8.80)	**< 0.001**	178 (70)	7.80 (6.15–11.38)	67 (63)	7.10 (4.92–10.68)	0.92	209 (75)	10.40 (7.60–15.00)	134 (75)	9.05 (6.03–15.04)	0.53
Transferrin (μg/L)	25.00–43.00	37 (3)	21.48 (17.34–23.87)	42 (4)	24.18 (19.72–28.11)	**0.034**	< 5		< 5			< 5		7 (4)	17.71 (16.04–26.88)	
**Coagulation**																
D-dimer (U/L)	< 0.65	345 (26)	1.10 (0.61–2.00)	245 (22)	0.71 (0.43–1.58)	**0.043**	83 (33)	1.40 (0.88–2.31)	22 (21)	0.82 (0.55–1.08)	**0.011**	70 (25)	1.74 (0.84–3.48)	40 (22)	1.38 (0.55–3.80)	0.85
International normalized ratio (INR)	0.80–1.20	888 (68)	1.10 (1.00–1.20)	754 (68)	1.00 (1.00–1.10)	**< 0.001**	187 (74)	1.05 (1.00–1.15)	66 (62)	1.02 (1.00–1.10)	0.32	217 (78)	1.10 (1.00–1.20)	141 (79)	1.05 (1.00–1.10)	0.062
Coagulation. tissue factor-induced	0.65–1.30	265 (20)	0.93 (0.79–1.06)	207 (19)	0.99 (0.85–1.17)	**< 0.001**	53 (21)	1.01 (0.87–1.11)	22 (21)	0.89 (0.83–1.06)	0.30	66 (24)	0.93 (0.80–1.10)	39 (22)	0.91 (0.84–1.14)	0.48
Partial thromboplastin time (s)	25.00–38.00	136 (10)	32.00 (24.50–38.25)	105 (9)	29.00 (24.50–35.00)	0.12	35 (14)	34.00 (27.00–37.00)	9 (8)	32.00 (25.00–33.00)	0.18	29 (10)	31.00 (26.00–36.00)	23 (13)	29.00 (27.00–38.50)	0.81
Fibrinogen (μmol/L)	5.40–10.90	60 (5)	15.50 (12.20–20.20)	49 (4)	13.30 (10.80–15.30)	**0.0064**	16 (6)	19.50 (15.02–21.93)	< 5			11 (4)	13.00 (11.30–16.95)	8 (4)	11.80 (9.85–14.55)	0.24
**Fluid and electrolyte balance**																
Sodium (mmol/L)	136–145	953 (72)	137.00 (134.67–139.50)	812 (73)	138.00 (135.25–140.00)	**0.026**	200 (79)	137.00 (134.00–139.04)	72 (68)	137.25 (134.30–139.00)	0.82	231 (83)	138.50 (135.00–142.00)	155 (87)	139.00 (135.50–142.00)	0.56
Potassium (mmol/L)	3.35–5.10	951 (72)	3.85 (3.56–4.10)	809 (72)	3.70 (3.50–4.00)	**0.0019**	200 (79)	3.85 (3.50–4.11)	72 (68)	3.79 (3.40–4.16)	1.00	230 (83)	3.93 (3.56–4.30)	154 (87)	3.90 (3.55–4.20)	0.73
Calcium ion (mmol/L)	1.18–1.37	481 (37)	1.17 (1.13–1.20)	385 (34)	1.19 (1.15–1.23)	**< 0.001**	103 (41)	1.14 (1.10–1.17)	42 (40)	1.15 (1.12–1.20)	0.063	129 (47)	1.16 (1.11–1.19)	88 (49)	1.18 (1.14–1.23)	**0.0072**
**Metabolism**																
Glucose (mmol/L)	2.90–7.80	748 (57)	7.00 (6.10–8.43)	657 (59)	6.53 (5.80–7.80)	**< 0.001**	156 (62)	7.50 (6.50–9.91)	56 (53)	7.48 (6.47–9.62)	0.74	187 (68)	7.25 (6.30–9.05)	124 (70)	6.80 (5.79–8.80)	**0.036**
HbA1c (mmol/L)	5.40–7.50	167 (13)	41 (36.5–50)	165 (15)	39 (36–45)	0.10	29 (11)	41.5 (37–64)	14 (13)	41 (36–54)	0.62	38 (14)	42 (36–48)	29 (16)	38 (34–42)	0.21
Triglyceride (mmol/L)	0.49–2.30	116 (9)	1.21 (0.91–1.62)	93 (8)	1.36 (1.05–1.88)	0.14	26 (10)	1.53 (1.20–2.00)	5 (5)	1.52 (1.36–1.54)	0.26	31 (11)	1.21 (0.99–1.58)	15 (8)	1.67 (1.15–2.39)	0.17
Albumin (g/L)	35–47	928 (71)	31.00 (27.00–34.50)	783 (70)	31.00 (27.50–35.00)	0.22	194 (77)	29.17 (25.50–33.38)	70 (66)	29.17 (26.00–33.00)	0.64	228 (82)	29.00 (25.00–33.00)	147 (83)	29.50 (24.75–32.50)	0.92

**Table 3:** Laboratory results from all COVID-19 hospitalised patients at admission, stratified on ICU admission and survival, as well as a comparison between biochemistry at hospitalisation for all patients and biochemistry at ICU admission for patients admitted to an ICU. Only the first contact contributed

Laboratory parameters (unit)	Median reference interval	Baseline biochemistry for not ICU admitted patients and ICU admitted patients					Baseline biochemistry for survivors and non-survivors					Baseline biochemistry at hospitalization and at ICU admission				
		Not ICU admitted ***n*** = 2072		ICU admitted ***n*** = 359			Survivors ***n*** = 1976		Non-Survivors ***n*** = 455			Hospitalisation ***n*** = 2431		ICU admission n = 359		
		n (%)	median (ICR)	n (%)	median (ICR)	p-value	n (%)	median (ICR)	n (%)	median (ICR)	p-value	n (%)	median (ICR)	n (%)	median (ICR)	p-value
**Acute phase reactants**																
C-reactive protein (mg/L)	< 7	1502 (72)	55,25 (24,00–105,00)	271 (75)	110,00 (54,75-182,00)	**< 0.001**	1381 (70)	55,50 (22,50-107,33)	392 (86)	83,50 (44,88-149,00)	**< 0.001**	1773 (73)	62,00 (27,50-116,00)	278 (77)	150,00 (100,00–246,75)	**< 0.001**
Ferritin (μg/L)	15–245	503 (24)	417,00 (189,50–910,50)	86 (24)	1018,50 (545,00–1907,50)	**< 0.001**	475 (24)	469,00 (199,00–1047,50)	114 (25)	596,50 (214,75-1269,25)	0.084	589 (24)	496,00 (200,00–1080,00)	128 (36)	1235,00 (721,25-2112,50)	**< 0.001**
Procalcitonin (μg/L)	< 0.30	262 (13)	0,10 (0,06-0,26)	79 (22)	0,43 (0,19-1,26)	0.25	282 (14)	0,12 (0,06-0,35)	59 (13)	0,33 (0,13-0,82)	0.65	341 (14)	0,14 (0,06-0,41)	147 (41)	0,67 (0,27-1,92)	**0.038**
Erythrocyte sedimentation rate (mm)	2.0–17.5	6 (0)	44,00 (28,75-55,50)	< 5			7 (0)	37,00 (19,50-54,00)	< 5			7 (0)	37,00 (19,50-54,00)			
Haptoglobin (g/L)	0.35–2.05	16 (1)	2,39 (1,62-3,78)	6 (2)	1,70 (1,62-2,88)	0.46	20 (1)	1,87 (1,60-3,78)	< 5			22 (1)	1,92 (1,61-3,68)	12 (3)	3,10 (1,50-4,48)	0.51
**Haematology**																
Haemoglobin (mmol/L)	6.60–9.65	1495 (72)	8,00 (7,20-8,80)	275 (77)	8,20 (7,40-8,85)	0.23	1386 (70)	8,10 (7,40-8,82)	384 (84)	7,77 (6,69-8,54)	**< 0.001**	1770 (73)	8,05 (7,25-8,80)	279 (78)	7,72 (6,85-8,37)	**< 0.001**
White blood cell count (× 10^9^/L)	4.45–13.30	1497 (72)	6,37 (4,80-8,64)	272 (76)	6,95 (5,19-9,96)	0.12	1379 (70)	6,23 (4,71-8,51)	390 (86)	7,30 (5,35-10,20)	**0.0027**	1769 (73)	6,45 (4,85-8,90)	280 (78)	8,89 (6,03-12,04)	**< 0.001**
Lymphocyte count (× 10^9^/L)	1.20–4.00	1379 (67)	1,00 (0,70-1,40)	244 (68)	0,83 (0,60-1,10)	**< 0.001**	1275 (65)	1,00 (0,74-1,40)	348 (76)	0,85 (0,60-1,20)	**0.037**	1623 (67)	1,00 (0,70-1,40)	231 (64)	0,80 (0,60-1,10)	**< 0.001**
Neutrophil count (× 10^9^/L)	1.60–7.10	1090 (53)	4,52 (3,20-6,61)	190 (53)	5,63 (3,94-8,40)	**0.0015**	1003 (51)	4,50 (3,20-6,38)	277 (61)	5,55 (3,85-8,56)	**< 0.001**	1280 (53)	4,70 (3,30-6,90)	187 (52)	7,50 (5,13-9,95)	**< 0.001**
Monocyte count (× 10^9^/L)	0.20–1.20	1379 (67)	0,50 (0,35-0,70)	244 (68)	0,40 (0,25-0,60)	**< 0.001**	1275 (65)	0,50 (0,30-0,70)	348 (76)	0,50 (0,30-0,74)	0.23	1623 (67)	0,50 (0,30-0,70)	231 (64)	0,33 (0,20-0,51)	**< 0.001**
Basophil count (× 10^9^/L)	0.00–0.20	1379 (67)	0,00 (0,00-0,05)	244 (68)	0,00 (0,00-0,05)	0.70	1275 (65)	0,00 (0,00-0,05)	348 (76)	0,00 (0,00-0,03)	0.16	1623 (67)	0,00 (0,00-0,05)	231 (64)	0,00 (0,00-0,05)	0.57
Platelet count (× 10^9^/L)	145–555	1462 (71)	208,50 (162,38-269,00)	272 (76)	200,00 (151,38-266,00)	0.086	1355 (69)	208,75 (164,00–269,75)	379 (83)	200,00 (149,75-263,33)	**0.044**	1734 (71)	207,33 (160,50-269,00)	279 (78)	228,00 (178,67-301,00)	**0.0023**
**Organ-specific markers**																
Alanine transaminase (U/L)	8–45	1388 (67)	27,50 (19,00-44,50)	255 (71)	34,00 (23,50-57,83)	**0.013**	1290 (65)	29,00 (20,00-48,00)	353 (78)	26,00 (18,00-41,50)	0.28	1643 (68)	28,50 (19,00-46,00)	259 (72)	40,25 (27,00-67,00)	**0.0038**
Aspartate transaminase (U/L)	15–40	50 (2)	35,00 (24,25-49,75)	20 (6)	58,50 (41,00–73,50)	0.066	61 (3)	42,00 (27,00-57,00)	9 (2)	29,00 (28,00–72,00)	0.94	70 (3)	41,50 (27,25-60,75)	59 (16)	62,00 (38,75-94,75)	0.065
Bilirubin (μmol/L)	3.40–21.50	1395 (67)	8,00 (6,00–11,00)	255 (71)	9,00 (7,00–13,00)	**0.021**	1292 (65)	8,00 (6,00–11,00)	358 (79)	9,00 (6,00–12,00)	0.23	1650 (68)	8,00 (6,00–11,50)	255 (71)	10,57 (7,50-14,83)	**0.0018**
Amylase (U/L)	25–112.5	697 (34)	58,00 (43,00–80,33)	132 (37)	54,75 (40,00–80,25)	0.77	640 (32)	57,00 (42,00–78,13)	189 (42)	63,00 (42,50–92,00)	**0.026**	829 (34)	57,00 (42,00–80,33)	116 (32)	59,00 (40,00-93,88)	0.064
Alkaline phosphatase (U/L)	50–284	1328 (64)	72,25 (57,50–95,00)	228 (64)	66,75 (52,00-97,63)	0.67	1214 (61)	69,00 (56,00-92,00)	342 (75)	79,00 (60,00–114,50)	**< 0.001**	1556 (64)	72,00 (57,00-96,00)	228 (64)	76,50 (57,00–128,50)	**0.0093**
Lactate dehydrogenase (U/L)	138–271	1278 (62)	255,00 (200,00–331,00)	228 (64)	388,50 (283,75-540,25)	**< 0.001**	1185 (60)	261,00 (201,00–347,00)	321 (71)	296,00 (230,00-421,00)	**< 0.001**	1506 (62)	269,00 (206,00–360,00)	249 (69)	483,50 (345,50-610,00)	**< 0.001**
Troponin I (ng/L)	< 45	128 (6)	15,00 (8,45-35,25)	36 (10)	28,50 (13,69-60,38)	0.92	137 (7)	15,00 (8,30-33,90)	27 (6)	47,00 (22,00–70,25)	0.11	164 (7)	17,50 (8,98-42,38)	58 (16)	32,95 (13,80-72,40)	0.14
Creatine kinase [muscle brain] (μg/L)	0.00–5.50	90 (4)	1,00 (1,00-2,00)	19 (5)	1,50 (1,00-3,00)	0.69	87 (4)	1,00 (1,00-2,00)	22 (5)	2,15 (1,08-4,88)	**0.018**	109 (4)	1,00 (1,00-2,30)	32 (9)	1,85 (1,00-3,88)	0.17
Creatine kinase (U/L)	40–260	208 (10)	105,25 (58,75-261,25)	36 (10)	218,75 (124,00–747,00)	0.080	195 (10)	118,00 (60,50-298,25)	49 (11)	147,00 (66,00–272,00)	0.40	244 (10)	124,00 (63,25-294,63)	51 (14)	183,00 (114,50-750,00)	0.19
Thyroid-stimulating hormone (10^−3^ IU/L)	0.55–4.65	314 (15)	1,24 (0,72-2,00)	50 (14)	0,96 (0,61-1,55)	0.063	291 (15)	1,13 (0,71-1,90)	73 (16)	1,31 (0,70-1,95)	0.52	364 (15)	1,15 (0,70-1,90)	41 (11)	1,10 (0,66-1,41)	**0.030**
Creatinine (μmol/L)	36.50–62.00	1269 (61)	80,00 (64,00–104,00)	220 (61)	94,50 (77,00–121,44)	**< 0.001**	1141 (58)	78,50 (63,67-99,00)	348 (76)	100,00 (75,88-142,58)	**< 0.001**	1489 (61)	82,00 (65,50-108,50)	224 (62)	90,50 (68,33-120,71)	**0.0027**
Glomerular filtration rate (estimated) (mL/min)	> 59.50	1492 (72)	76,00 (54,00-90,00)	273 (76)	66,00 (51,50-85,00)	**0.0013**	1373 (69)	79,50 (59,50–90,00)	392 (86)	54,75 (35,46-73,13)	**< 0.001**	1765 (73)	75,00 (53,50-89,00)	282 (79)	72,75 (52,63-89,19)	0.41
Urea (mmol/L)	2.60–7.50	1314 (63)	6,10 (4,21-9,30)	245 (68)	7,80 (5,85–10,90)	**< 0.001**	1216 (62)	5,70 (4,05-8,10)	343 (75)	9,80 (7,00–15,00)	**< 0.001**	1559 (64)	6,30 (4,40-9,60)	256 (71)	7,14 (5,10–10,46)	0.14
Transferrin (μg/L)	25.00–43.00	75 (4)	22,61 (18,84-27,64)	< 5			68 (3)	22,61 (18,84-27,64)	11 (2)	22,00 (17,00–26,88)	0.47	79 (3)	22,61 (18,84-27,51)	11 (3)	16,96 (13,76-18,84)	**< 0.001**
**Coagulation**																
D-dimer (U/L)	< 0.65	485 (23)	0,84 (0,47-1,80)	105 (29)	1,30 (0,82-2,00)	0.16	480 (24)	0,84 (0,49-1,60)	110 (24)	1,59 (0,82-3,58)	**0.014**	590 (24)	0,90 (0,51-1,87)	149 (42)	1,68 (1,08-3,81)	**< 0.001**
International normalized ratio (INR)	0.80–1.20	1389 (67)	1,05 (1,00-1,10)	253 (70)	1,05 (1,00-1,13)	0.88	1284 (65)	1,04 (1,00-1,10)	358 (79)	1,10 (1,00-1,20)	**0.0054**	1642 (68)	1,05 (1,00-1,10)	266 (74)	1,10 (1,00-1,17)	0.21
Coagulation. tissue factor-induced	0.65–1.30	397 (19)	0,95 (0,82-1,12)	75 (21)	0,98 (0,85-1,10)	0.34	367 (19)	0,96 (0,82-1,11)	105 (23)	0,92 (0,82-1,11)	0.82	472 (19)	0,95 (0,82-1,11)	84 (23)	0,92 (0,78-1,02)	0.051
Partial thromboplastin time (s)	25.00–38.00	197 (10)	29,00 (24,50-37,00)	44 (12)	32,50 (26,50-35,63)	0.83	189 (10)	31,00 (24,00–36,00)	52 (11)	30,50 (26,75-38,00)	0.78	241 (10)	31,00 (24,50-37,00)	54 (15)	34,00 (27,00–38,75)	0.12
Fibrinogen (μmol/L)	5.40–10.90	93 (4)	13,50 (11,45-16,90)	16 (4)	19,50 (15,02-21,93)	**0.0041**	90 (5)	14,20 (12,10–19,09)	19 (4)	13,00 (10,10–15,20)	**0.038**	109 (4)	14,10 (11,90-18,50)	41 (11)	18,60 (13,00–20,80)	**0.027**
**Fluid and electrolyte balance**																
Sodium (mmol/L)	136–145	1493 (72)	137,50 (135,00–140,00)	272 (76)	137,00 (134,00–139,00)	**0.010**	1379 (70)	137,33 (135,00–139,33)	386 (85)	138,92 (135,00–142,00)	**< 0.001**	1765 (73)	137,50 (135,00–140,00)	275 (77)	138,77 (135,73-141,67)	**< 0.001**
Potassium (mmol/L)	3.35–5.10	1488 (72)	3,80 (3,50-4,07)	272 (76)	3,81 (3,45-4,14)	0.22	1376 (70)	3,75 (3,50-4,03)	384 (84)	3,92 (3,55-4,28)	**< 0.001**	1760 (72)	3,80 (3,50-4,07)	274 (76)	3,83 (3,60-4,10)	**0.023**
Calcium ion (mmol/L)	1.18–1.37	721 (35)	1,18 (1,14-1,22)	145 (40)	1,15 (1,11-1,18)	**< 0.001**	649 (33)	1,18 (1,14-1,21)	217 (48)	1,17 (1,12-1,21)	0.21	866 (36)	1,17 (1,14-1,21)	159 (44)	1,14 (1,10-1,18)	**< 0.001**
**Metabolism**																
Glucose (mmol/L)	2.90–7.80	1193 (58)	6,70 (5,90-7,95)	212 (59)	7,50 (6,50-9,74)	**< 0.001**	1094 (55)	6,70 (5,98-7,90)	311 (68)	7,00 (6,10-8,94)	**0.0029**	1405 (58)	6,80 (6,00-8,20)	166 (46)	8,08 (6,73-10,74)	**< 0.001**
HbA1c (mmol/L)	5.40–7.50	289 (14)	6,55 (6,00-7,60)	43 (12)	6,90 (6,20-9,35)	**0.049**	265 (13)	6,60 (6,00-7,70)	67 (15)	6,50 (5,95-7,60)	0.59	332 (14)	6,60 (6,00-7,70)	33 (9)	7,10 (6,40-7,60)	0.10
Triglyceride (mmol/L)	0.49–2.30	178 (9)	1,21 (0,96-1,80)	31 (9)	1,52 (1,20-1,89)	0.22	163 (8)	1,25 (0,96-1,80)	46 (10)	1,28 (1,00-1,80)	0.39	209 (9)	1,26 (0,97-1,80)	61 (17)	1,82 (1,41-2,10)	**< 0.001**
Albumin (g/L)	35–47	1447 (70)	31,00 (27,50-35,00)	264 (74)	29,17 (25,67-33,00)	**< 0.001**	1336 (68)	31,00 (27,67-35,00)	375 (82)	83,50 (44,88-149,00)	**< 0.001**	1711 (70)	31,00 (27,00–34,50)	269 (75)	25,00 (21,67-29,00)	**< 0.001**

Multiple laboratory systems are used in the Danish healthcare system to analyze biochemical samples. These systems may be used with model adapted reference ranges. In this table the reference ranges are medians of all the reporting laboratories and as a result the reference ranges are only intended as interpretation assistance. Groups with less than five patients are presented as < 5. P-values< 0.05 are bold.

### Mortality

**Fig. 1:**
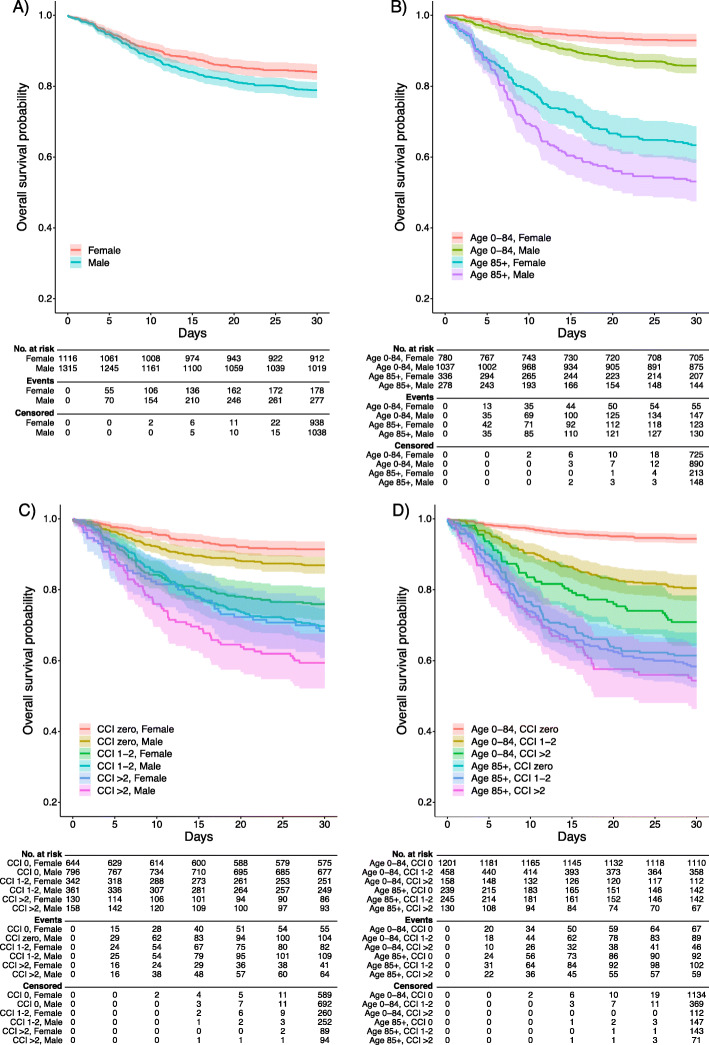
Kaplan-Meier curves illustrating 30-day survival. A) Sex. B) Sex and age. C) Charlson comorbidity index modified by Quan and sex. D) Charlson comorbidity index modified by Quan and age-group

During follow-up, 455 (18.7%) hospitalised patients died, 277 (60.9%) of these were males. The 30-day mortality among patients admitted to an ICU was 107 (29.8%) (Table 1). The 30-day KM survival rate was 81.2% (95% CI: 79.7–82.8). This was lower for males than for females (78.9% [95% CI: 76.7–81.1%] and 84.0% [95% CI: 81.9–86.2%] respectively, *p* < 0.01). Patients with higher CCI index had lower 30-day survival rates with 63.3% (95% CI: 58.0–69.2%) for those with a CCI > 2 and 88.9% (CI: 87.3–90.6%) for CCI of zero. The effect of the level of CCI was more pronounced in patients of older age (Fig. 1).

### ICU admission

Among all 2431 hospitalised patients included in the study, 359 (14.8%) were admitted to an ICU (median age 69 years [IQR 59–75], 70.5% male), 240 (66.9%) required IMV, 78 (21.7%) dialysis, 210 (58.5%) vasopressors and 18 (5%) ECMO (Table 1). The cumulative incidence rate of ICU admission during the first week after hospitalisation was 12.6% (95% CI: 11.3–13.9%), the risk was lower for females than males (7.9% vs. 16.7%, *p* < 0.001) and for patients aged 85 and older compared to those under 85 (5.4% vs. 15.1%, *p* < 0.001). There was no difference on CCI with 12.7% (95% CI: 11.0–14.4%), 13.7% (95% CI: 11.1–16.2%) and, 9.7% (95% CI: 6.3–13.1%) for CCI zero, 1–2 and > 2 respectively (*p* = 0.601). The cumulative incidence of ICU admission only increased slightly after the first 7 days of follow-up.

### Prognostic factors for death

**Fig. 2:**
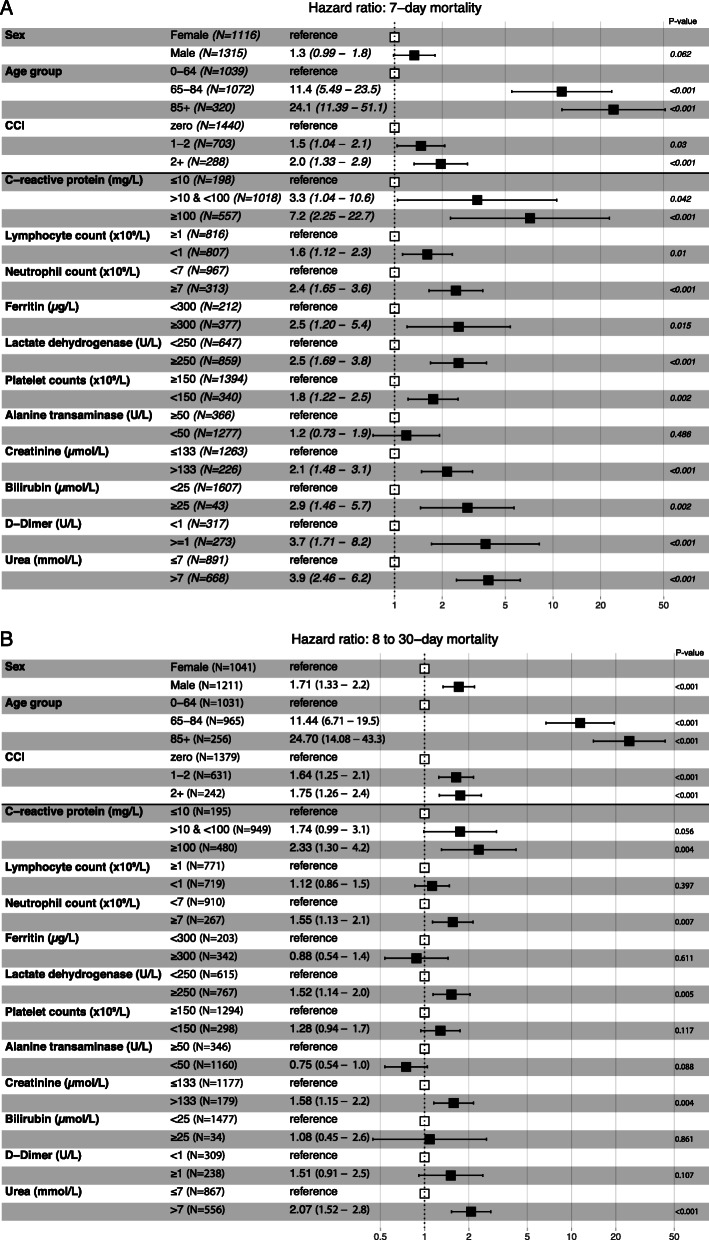
**a** and **b** Forest plot showing adjusted hazard ratios obtained by multivariate Cox regression analysis of prognostic factors of death in patients admitted with Covid-19. Patients who died during the first 7-days after admission were excluded from the analyses of 8 to 30-day mortality. Models were adjusted for sex, age-group, and Charlson Comorbidity Index (0, 1–2, > 2). Hazard ratio’s for laboratory measure were each adjusted individually for sex, age-group and CCI. Where noted, the biometrics were entered on the log2 scale. Reference: normal value; CI: confidence interval; HR: hazard ratio

In the multivariate analyses (Fig. 2 a and b), the strongest independent risk factor for death within both days 1–7 and 8–30 was age. Conditional upon surviving the first week, the underlying comorbidity level, increasing age and male sex were all independently associated with death during day 8–30. All explored abnormalities in baseline laboratory parameters at admission were independently associated with death within the first 7 days except alanine transaminase, whereas elevated CRP, neutrophil count, LDH, creatinine and urea were independently associated with death within 30-days given survival of the first week.

## Discussion

In this nationwide study of COVID-19 patients, 2431 individuals, of which 60.7% had a registered comorbidity within the past 10 years, were hospitalised. ICU admission was required in 14.8% of the hospitalised individuals. Advanced age, high levels of systemic inflammation (CRP, ferritin, D-dimer), high cell turnover (LDH), azotaemia (urea), kidney function (creatinine), and haemato-lymphoid reaction (lymphopenia, neutrophilia, thrombocytopenia) were independently associated with death within the first week of admission. Conditional upon surviving the first week, male sex, age, comorbidity, elevated CRP, LDH, creatinine, urea and neutrophilia were independently associated with death within 8–30 days. Patients who later required ICU admission had higher markers of inflammation, cell-turnover and metabolic dysregulation.

Our findings are in line with the previously reported effects of age, male sex, and level of comorbidities on both mortality and the need for ICU admission [1, 3, 7, 17, 24]. In contrast to previous reports [1, 2, 16], a remarkably high proportion (nearly two thirds of patients) had low levels of comorbidity preceding the COVID-19 admission. Having a moderate to high level of comorbidity was associated with a nearly two-fold increase in mortality. Males and females had similar mortality rates during the first week of admission, but likelihood for death significantly diverged towards an increased fatality rate among males after surviving the first week for all analysed strata. The reasons behind these differences in early and late mortality are not clear, but probably multifactorial. Sex differences in thromboembolic events could be a residual confounder of clinical relevance which may explain a difference between 7-day and 30-day mortality for males and females.

From a clinical point of view, it is reasonable to assume, that the immediate time after hospitalization may be more critical where sex differences play less of a role and the acute state of the infection is more relevant. Thereafter other factors may become relevant as the acute threat subsides. The acute phase may be more pronounced at time of hospital admission and is stronger reflected in the baseline laboratory findings but become less relevant for the final outcome. We do cease the point that the cut-off time of 7 days and 30 days are relatively arbitrary, although they do align with an observation for worsening clinical outcomes in our hospitalised patients.

In line with previous findings, patients who were at highest risk of ICU admission were males with higher CRP, LDH and blood glucose among others, indicating higher levels of inflammation, cell-turnover and metabolic dysregulation [3, 7, 14, 15]. Sex differences in laboratory parameters were markedly less pronounced in the subgroup of patients who presented with more severe outcomes, including ICU admission and death. Differences in the degree of inflammatory response between males and females could be a field for future research.

Abnormal coagulation parameters on admission, including D-dimer, were associated with a higher risk of death but not ICU admission (Table 3) as shown in other studies [7, 17]. This could be related to a low number of events among patients where the test was taken.

### Study strengths and limitations

Since Danish health care registries are independently recorded and complete [19–21], the risk of selection bias and loss to follow-up was minimal. However, we acknowledge potential differences in referral patterns during the epidemic. Moreover, during the early-stage of the epidemic, treatment of COVID-19 was merely symptomatic. By early April 2020, a few pharmacological interventions were available mostly within the frame of clinical trials (https://laegemiddelstyrelsen.dk/en/news/2020/covid-19-see-the-list-of-approved-danish-clinical-trials-of-medicines-for-covid-19/), which, alongside with improved clinical management, could have had an impact on our outcome estimates over time. In comparison to previous studies from Denmark [5, 6] we found a higher mortality. However, we report outcomes in a selected group of patients namely those in need of hospitalisation and therefore presenting with a more severe clinical course.

The diagnosis of COVID-19 and several other variables have not yet undergone full internal validation to date. In our survival analysis, loss to follow-up in relation to the primary outcome (mortality) could cause informative censoring. However, as borders were closed during most of the study period, we consider this problem minimal. Also, we did not have access to vital parameters (blood pressure, oxygen saturation etc.) through the data sources.

Although speculative, the massive lock-down, and organisational changes with extraordinary resources allocated could in part explain the possible lower median hospital duration compared to other studies [25]. Moreover, information on the actual viral load and shredding could be an indicator for the state of infection at hospitalisation but was not available in the registers at the time of this study. Sex differences in care-seeking behaviour with males presenting with a more advanced stage of disease is also a possibility.

Studies of diseases and conditions that may develop rapidly with fatal outcome can be susceptible to Neyman bias, which arises when a gap in time occurs between exposure and selection of study participants [26]. E.g. if a sizable number of individuals arrived at the hospital but died before COVID-19 testing was in place, this bias may apply. We addressed this bias by using the first admission date to the hospital as the start of follow-up rather than the date of diagnosis. This first admission was identified by iteratively looking back for any hospital contacts within 24 h previously.

Regarding missing data, we argue that laboratory parameters are missing in a systematic, non-random fashion as blood samples were taken only when a healthcare worker deemed it appropriate based on a clinical judgment. The adjusted hazard ratios should thus be interpreted as prognostic factors for a highly selected population for which laboratory parameters were relevant for clinical reasons [27]. Of the 2431 patients included, 1999 (82%) had laboratory results taken ±24 h upon admission. The majority of cases have been seen in the capital region (www.sst.dk/corona). The register we used (RLRR) covers the entire country except Midtjylland Region. We had 263 admitted patients from this region who did not have laboratory results registered due to the lack of synchronization with data from this region. Moreover, 169 patients in the Capital Region (Copenhagen) have missing laboratory results. In the initial phase of the epidemic, all patients in the Capital Region with a positive SARS-CoV-2 test were admitted regardless of severity of the disease. We argue that although the laboratory results pertain to a group of patients with severe symptoms, they may well be indicative of factors of importance in the clinical follow-up and outcome of severely ill COVID-19 patients.

We chose to use baseline laboratorial values at admission and their effect on outcome, since important clinical decisions on observation and management often are made at this time. We therefore did not analyse the trend dynamics of the covariates used or the effect of higher values during the course of admission, which would require other regression modelling and is a different research question altogether beyond the scope of the present article. Not all individuals had laboratory results for all of the parameters upon admission whereby adjusting missing values with interpolation methods on such a highly selected population were not deemed appropriate. Thus, we choose to report on the laboratory findings individually adjusting only for age, sex and CCI.

Our analysis show that adjusted for pre-existing factors, several of the laboratory findings are related to increased mortality risk. However, we acknowledge that the baseline laboratory markers are different from other pre-existing determinants, and therefore could be directly on the causal pathway to more severe clinical disease or death. Including both may have resulted in an underestimate of the importance of laboratory values and may partially mask the indirect influence of more distal factors like sex.

The laboratory cut-off parameters for the multivariate analysis were arbitrary selected by the investigators. This could perhaps have resulted in an underestimation of the association between some laboratory parameters and outcomes. We have therefore included detailed stratified description of the results in Tables 2 and 3, and the multivariate analysis should be interpreted in this context.

Finally, the generalizability of our results to other settings would be limited due to organizational and capacity differences between health systems in different countries, particularly regarding the definitions of ICU admissions.

## Conclusion

Among hospitalised patients during the first wave of COVID-19 in Denmark, advanced age, male sex, chronic comorbidities, and selected clinical and laboratory parameters were all independently associated with higher risk of severe COVID-19 related outcomes. Males presented with more pronounced laboratory abnormalities at hospital admission. Males and females had comparable mortality during the first week of admission, but the likelihood for death was significantly higher for males after the first week. ICU admitted patients had higher markers of inflammation, cell-turnover, and metabolic dysregulation. Identification of such factors can be used for planning strategies targeting specific high-risk individuals.

## Supplementary Information

**Additional file 1 Table S1**

## Data Availability

Public access to the databases used is closed but available upon request at the Danish Health Data Authority (https://sundhedsdatastyrelsen.dk/da/kontakt). Authors were granted administrative access to raw anonymised data used in the study at the Danish Health Data Authority by permission from the Danish Health and Medicines Authority (ID: 31–1521-263) and by the Danish Data Protection Agency (P-2020-375).

## Abbreviations

B972A: *COVID-19 severe acute respiratory syndrome*
B342A: *COVID-19 infection unspecified sites*
CCI: Charlson comorbidity index modified by Quan
CI: 95% confidence intervals
COVID-19: coronavirus disease 2019
CRP: C-reactive protein
CRS: Danish Civil Registration System
DNPR: Danish National Patient Register
ECMO: Extracorporeal membrane oxygenation
HR: Hazard ratios
ICU: Intensive care unit
ICD: International Classification of Diseases, 10th revision
IMV: Invasive mechanical ventilation
IQR: Interquartile range
LDH: Lactate dehydrogenase
KM: Kaplan-Meier
NPU: Nomenclature for Property and Unit
PCR: Polymerase Chain Reaction
RLRR: Register of Laboratory Results for Research
PRN: Personal Registration Number
SARS-CoV-2: Severe acute respiratory syndrome coronavirus 2
STROBE: STrengthening the Reporting of OBservational studies in Epidemiology
