# Supplementary material for: First wave of COVID-19 hospital admissions in Denmark: a Nationwide population-based cohort study
Source: BMC Infect Dis. 2021 Jan 9;21:39. doi: 10.1186/s12879-020-05717-w (PMC7794638; doi:10.1186/s12879-020-05717-w)
Supplement: Supplementary file 1 — Additional file 1 Table S1 [file 12879_2020_5717_MOESM1_ESM.docx]

Supplementary Table S1

| **Comorbidity or procedure** | **ICD-10 codes or SKS-codes*** |
| --- | --- |
| **Charlson Comorbidity Index** |  |
| Myocardial infarction | I21*, I22*, I25.2 |
| Congestive heart failure | I09.9, I11.0, I13.0, I13.2, I125.5, I42.0, I42.5-I42.9, I43*, I50*, P29.0 |
| Peripheral vascular disease | I70*, I71*, I73.1, I73.8, I73.9, I77.1, I79.0, I79.2, K55.1, K55.8, K55.9, Z95.8, Z95.9 |
| Cerebrovascular disease | G45*, G46*, H34.0, I60*-I69* |
| Dementia | F00*-F03*, F05.1, G30*, G31.1 |
| Chronic pulmonary disease | I27.8, I27.9, J40*-J47*, J60*-J67*, J68.4, J70.1, J70.3 |
| Connective tissue disease | M05*, M06*, M31.5, M32*-M34*, M35.1, M35.3, M36,0 |
| Ulcer disease | K25*-K28* |
| Mild liver disease | B18*, K70.0, K701-K70.3, K70.9, K71.3-K71.5, K71.7, K73*, K74*, K76.0, K76.2-K76.4, K76.8, K76.9, Z94.4 |
| Severe liver disease | I85.0, I85.9, I86.4, I98.2, K70.4, K71.2, K72.9, K76.5-K76.7 |
| Diabetes without organ damage | E10.0, E10.1, D10.6, E10.8, E10.9, E11.0, E11.1, E11.6, E11.8, E11.9, E12.0, E12.1, E12.6, E12.8, E12.9, E130, E13.1, E13.6, E13.8, E13.9, E14.0, E14.1, E14.6, E14.8, E14.9 |
| Diabetes with organ damage | E10.2-E10.5, E12.7, E11.2-E11.5, E11.7, E12.2-E12.5, E12.7, E13.-E13.5, E13.7, E14.2-E14.6, E14.7 |
| Hemiplegia | G04.1, G11.4, G80.1, G80.2, G81*, G82*, G83.0, G83.2-G83.4, G83.9 |
| Renal disease | I12.0, I13.1, N03.2-N03.7, N05.2-N05.7, N18*, N19*, N25.0, Z49.0, Z49.1, Z49.2, Z94.0, Z99.2 |
| Malignancy, including leukaemia and lymphoma | C00*-C26*, C30*-C34*, C37*-C41*, C43*, C45*-C58*, C60*-C76*, C81*-C85*, C88*, C90*-C97* |
| Metastatic solid tumour | C77*-C80* |
| HIV/AIDS | B20*-B22*, B24* |
|  |  |
| **Others** |  |
| Obesity | E66.0A, E66.0B, E66.0C, E66.0E-E66.0H, E66.1, E66.2, E65.8, E65.9, E66.8, E66.9, T98.3B, Z48.8D, Z71.8B2, |
| Neurological disease | M51*, G10*-G14*, G20*-G23*, G35*, G36.1-G36.9, G37*, G71*-G73*, G80*-G83*, G90.1, G90.2, G90.3-G90.9, G91*, G93*-G94*, G99* |
| Chronic Obstructive Pulmonary Disease | J40*-J44*, J47* |
| Asthma | J45*, J46* |
| Immunosuppression | B20*-B24*, O98.7, Z21*, C00*-C99*, D37*-D48* |
| Hypertension | I109*-I111* |
|  |  |
| **SKS ICU codes** |  |
| ICU treatment | NABB, NABE |
| Respirator use | BGDA0, BGDA1 |
| ECMO | BGXA2 |
| Vasopressor treatment | BFHC95, BFHC92, BFHC93 |
| Dialysis | BJFD0 |
|  |  |
